# Hierarchical government environmental confidence and willingness to pay for environmental protection: evidence from urban–rural China

**DOI:** 10.3389/fpubh.2026.1853242

**Published:** 2026-06-19

**Authors:** Xiaoxiang Yan, Shanshan Gong

**Affiliations:** 1 College of Humanities and Foreign Languages, China Jiliang University, Hangzhou, China; 2 The School of Law, Hangzhou City University, Hangzhou, China; 3 Guanghua Law School, Zhejiang University, Hangzhou, China

**Keywords:** China, environmental confidence, environmental governance, hierarchical government confidence, media use, urban–rural divide, willingness to pay

## Abstract

**Introduction:**

Public willingness to pay (WTP) for environmental protection is a key indicator of societal readiness to bear the costs of environmental governance. However, the role of citizens’ confidence in government environmental performance at different hierarchical levels remains largely unexplored. This study introduces the concept of hierarchical government environmental confidence into WTP research, examining how citizens’ differential evaluations of central versus local government environmental governance predict WTP.

**Methods:**

Using nationally representative data from the 2021 Chinese General Social Survey (CGSS2021, *N* = 1,638), we employed hierarchical multiple regression with heteroskedasticity-robust standard errors, urban–rural subgroup analysis, and multiple robustness checks.

**Results:**

Both central government environmental confidence (CGC, b = 0.062, *p* < 0.05) and local government environmental confidence (LGC, b = 0.105, *p* < 0.01) positively predict WTP, with LGC showing a numerically larger coefficient. A suggestive urban–rural divergence emerges: urban residents’ WTP is more closely associated with local government confidence, whereas rural residents’ WTP is more closely associated with central government confidence. New media use negatively predicts WTP, while traditional media use shows no significant effect. Government confidence variables explain substantially more WTP variance than media use variables.

**Discussion:**

These findings highlight the importance of hierarchical institutional confidence in shaping public environmental investment and offer differentiated policy implications for urban and rural environmental governance.

## 1 Introduction

Environmental degradation remains one of the most pressing challenges confronting developing nations in their pursuit of sustainable development. China, as the world’s largest developing country, has experienced severe ecological consequences alongside its remarkable economic growth (1). Public participation in environmental governance has emerged as a critical policy area, with the Chinese government emphasizing multi-actor collaborative environmental governance (2). Recent evidence suggests that China’s environmental policy instruments, such as environmental tax reform, have effectively promoted green technological innovation at the firm level (3). Among various forms of public engagement, willingness to pay (WTP) for environmental protection serves as a precise indicator of the public’s readiness to bear economic costs to improve environmental quality (4, 5). Understanding the factors that shape public WTP is therefore essential for designing effective environmental policies and mobilizing societal resources.

Considerable scholarly attention has been directed toward identifying the determinants of environmental WTP. Sociodemographic characteristics such as income, education, and gender have been consistently identified as significant predictors (6, 7). Ren et al. (8) found that gender equality awareness mediates the relationship between gender and WTP in China. Fan and An (9) revealed a paradox whereby higher socioeconomic status predicts more environmental actions but lower WTP. From the communication perspective, Wang (10) demonstrated that traditional media use enhances WTP through cultivating environmental responsibility rather than environmental knowledge. Meanwhile, Sun and Kong (11) found that new media usage and media trust negatively impact WTP through a “clicktivism” mechanism. Despite these advances, one crucial dimension remains largely unexplored in the WTP literature: the role of public confidence in government environmental governance at different hierarchical levels.

Government trust constitutes a fundamental psychological foundation for public participation in collective action and public goods provision (12). In the Chinese context, research on government trust has revealed a distinctive pattern known as “hierarchical government trust.” (*chaoxu zhengfu xinren*). This pattern refers to the phenomenon wherein citizens consistently express substantially higher trust in the central government than in local governments (13, 14). Li (15) found that while many villagers trust the central government’s intent, they distrust its capacity to ensure faithful policy implementation at local levels. Subsequent research has identified multiple sources of this hierarchical pattern, including the semi-open media environment, cultural orientations toward authority, and dissatisfaction with local public service delivery (16). Xu et al. (17) further demonstrated that traditional media usage is positively associated with trust in the central government but unrelated to local government trust, while social media usage is negatively associated with local government trust but not with central government trust. Yet, despite its theoretical significance, the concept of hierarchical government trust has rarely been applied to the study of environmental WTP.

Existing studies linking government trust to environmental outcomes have generally treated government trust as a unidimensional construct. Hu et al. (18) examined the mediating role of government trust between media use and environmental public service satisfaction, but did not differentiate between central and local government trust. Geng and He (19) investigated how environmental regulation affects environmental governance satisfaction, finding that environmental awareness negatively moderates this relationship. Tan et al. (20) confirmed that institutional trust and interpersonal trust both positively affect environmental WTP, yet their analysis did not consider the hierarchical nature of Chinese citizens’ evaluations of different government levels. Similarly, Jia and Lin (21) demonstrated that public satisfaction with government environmental performance significantly promotes environmental participation, but their analysis likewise treated government performance evaluation as a unidimensional construct without distinguishing central and local levels. Anderson (22) found that trust in government institutions generally has a significant positive effect on WTP for public goods in transition countries. However, this undifferentiated treatment obscures the fact that in China’s multi-level governance system, the central government formulates environmental policies while local governments implement them, and citizens’ evaluations of these two levels may have distinct implications for WTP.

Furthermore, the potential heterogeneity of the government confidence–WTP nexus across urban and rural contexts has received insufficient attention. China’s urban–rural divide constitutes a fundamental structural feature that shapes environmental attitudes and behaviors (23). Yu (24) found that rural Chinese are less concerned about the environment than their urban counterparts, partly due to limited education and information access. Cheng and Mao (25) demonstrated significant urban–rural disparities in environmental behavior associated with socioeconomic status differences. Ding and Wang (26) found that urban and rural residents’ WTP for green energy consumption diverges substantially, with capital endowment contributing far more to this gap than energy cognition. Given these systematic differences, it is plausible that the pathways through which government environmental confidence influences WTP vary across urban and rural settings. However, no existing study has examined this possibility.

A third gap concerns the independent explanatory power of media use for WTP after accounting for government environmental confidence. While extensive research has demonstrated media effects on environmental attitudes (10, 11, 27), the question of whether these effects remain significant when government trust dynamics are controlled has not been addressed. Miao et al. (28) found that political trust mediates the relationship between media use and political participation in China. Gong et al. (29) showed that media credibility mediates the association between media usage and political trust among young Chinese adults. These findings suggest that much of what has been attributed to ‘media effects’ on environmental behavior may operate through the government trust channel, but this possibility has not been empirically tested in the WTP domain.

To address these gaps, this study proposes a dual-pathway model examining how media use and hierarchical government environmental confidence jointly predict WTP among Chinese residents. Drawing on data from the 2021 Chinese General Social Survey (CGSS2021), a nationally representative dataset of 8,148 respondents, we employ hierarchical regression analysis to disentangle the independent contributions of these two pathways, and further investigate urban–rural heterogeneity through subgroup analysis.

This study makes three contributions. First, it introduces the concept of ‘hierarchical government environmental confidence’ into the WTP research domain, distinguishing between central and local government environmental governance evaluations and testing their differential effects. Our findings reveal that local government environmental confidence (LGC, *b* = 0.105, *p* < 0.01) shows a numerically larger coefficient than central government environmental confidence (CGC, *b* = 0.062, *p* < 0.05). Second, it provides suggestive evidence of urban–rural heterogeneity in the confidence–WTP nexus. Specifically, urban residents’ WTP is more closely associated with local government confidence, whereas rural residents’ WTP is more closely associated with central government confidence, though interaction tests suggest this pattern should be interpreted cautiously. This pattern reflects distinct governance experiences across the urban–rural divide and offers implications for differentiated environmental mobilization strategies. Third, it suggests that, under the current operationalization, government environmental confidence accounts for substantially more WTP variance than media use frequency, indicating that previous media effect findings may be partially confounded with government confidence dynamics.

The remainder of this paper is organized as follows. Section 2 reviews the literature and develops the research hypotheses. Section 3 describes the data, variables, and analytical strategy. Section 4 presents the empirical results, including hierarchical regression, subgroup analysis, and robustness checks. Section 5 discusses the theoretical and practical implications. Section 6 concludes with a summary, policy recommendations, and limitations.

## 2 Literature review and hypotheses development

### 2.1 Government trust and willingness to pay for environmental protection

Environmental protection is fundamentally a public goods problem: individuals are asked to contribute private resources toward collective environmental improvement, with the expectation that government institutions will effectively deploy these resources (2, 12). This logic places government trust at the center of the WTP calculus. When citizens trust that government agencies will use environmental funds efficiently, they are more willing to bear costs; conversely, distrust may reduce WTP by generating skepticism about the efficacy of personal contributions (20, 22).

Empirical evidence broadly supports this expectation. Anderson (22), analyzing European transition countries, found that trust in government institutions generally has a significant positive effect on WTP for public goods including environmental protection. Tan et al. (20), using CGSS2021 data, confirmed that institutional trust positively affects environmental WTP, with environmental self-efficacy partially mediating this relationship. In the Chinese context, Geng and He (19) found that environmental regulation positively influences environmental governance satisfaction, but this relationship weakens when public environmental awareness is high, suggesting that more aware citizens hold government performance to higher standards. Hu et al. (18) confirmed that government trust mediates the relationship between traditional media use and environmental public service satisfaction. Wu and Cao (30) demonstrated that ecological environment satisfaction significantly enhances residents’ happiness, implying that subjective perceptions of government environmental performance have tangible well-being consequences.

However, a critical limitation of existing research is its treatment of government trust as monolithic. In China’s multi-level governance system, environmental policy is formulated at the central level but implemented primarily by local governments. The central government sets targets, enacts legislation, and conducts supervisory inspections, while local governments bear the primary responsibility for day-to-day pollution monitoring, emission control, and ecological restoration (31, 32). This vertical division of labor means that citizens’ evaluations of central and local government environmental performance may diverge substantially, with different implications for WTP.

### 2.2 Hierarchical government trust in the Chinese context

The concept of ‘hierarchical government trust’ captures a well-documented pattern in Chinese political attitudes: citizens express substantially higher trust in the central government than in local governments (13–15, 33). Li’s (15) seminal work on rural China showed that while villagers trust the central government’s intent, they distrust its capacity to ensure faithful policy implementation, and this combination may encourage ‘rightful resistance’ against local officials in the name of the center. Lu (14) systematically conceptualized hierarchical government trust (*chaoxu zhengfu xinren*) and identified its determinants using three waves of Asian Barometer data, finding that the semi-open media environment, moderate authoritarian cultural orientation, and public dissatisfaction with grassroots public services jointly generate and reinforce this phenomenon.

Subsequent research has elaborated the mechanisms and consequences of this hierarchical pattern. Wu et al. (34) found that authoritarian values, economic performance, and government responsiveness all positively influence both central and local trust, but authoritarian values and government responsiveness are significant sources of the trust gap itself. Chen and Zhu (35) identified five factors driving hierarchical trust through grounded theory analysis, including perceived performance gaps between central and local governments across domains such as economic development, public services, and environmental protection. Zhai (36) demonstrated that the central government loses public trust specifically due to unsatisfying economic performance, while local governments risk losing trust primarily for corruption, and both levels lose trust for poor environmental protection performance. Huang (37) showed that Chinese citizens tend to blame local governments for problems, whereas Taiwanese citizens are inclined to criticize the central government, reflecting different information environments and attribution patterns.

Of particular relevance to this study, research on media and hierarchical trust has demonstrated differential effects across media types and government levels. Xu et al. (17), using surveys from 2013 and 2018, found that traditional media usage is positively associated with central government trust but unrelated to local government trust, while social media usage is negatively associated with local government trust but not central government trust. Meng and Li (38) found that news consumption effects on hierarchical political trust are mediated by perceived happiness and moderated by authoritarian values. Chen (39) found that citizens who use the Internet more tend to trust the central government less, while those who perceive high quality of public services tend to trust their local governments more. Li’s (40) machine learning analysis of a national survey revealed that 85% of respondents appear to trust the central government at first glance, but upon deeper analysis only 18% have total trust, 34% have partial trust, and 33% are skeptical.

We use the term “environmental confidence” rather than “trust” to capture citizens’ evaluative judgments about government environmental governance performance. In the Chinese political context, citizens’ assessments of government performance in specific policy domains serve as the primary empirical expression of political confidence (13, 36). As Zhai (36) demonstrated, policy performance evaluations directly shape central–local political trust. Our measures (H8 and H11) capture citizens’ confidence in government environmental governance effectiveness based on their evaluations of past performance, which we conceptualize as the experiential foundation of domain-specific institutional confidence. We do not equate this with diffuse political trust; rather, it represents a performance-based evaluative judgment specific to the environmental governance domain.

We argue that this hierarchical confidence pattern should produce differential effects on environmental WTP. Central government environmental confidence (CGC) reflects citizens’ assessment of the national strategic direction on environmental issues. This macro-level confidence may enhance WTP by generating a sense of systemic legitimacy. Local government environmental confidence (LGC) reflects whether environmental investments are being effectively implemented on the ground. This implementation-level confidence may be even more directly consequential for WTP, because citizens’ personal environmental expenditures are ultimately channeled through local governance structures. Based on this reasoning:

*Hypothesis 1 (H1): Central government environmental confidence (CGC) positively predicts willingness to pay for environmental protection.*

*Hypothesis 2 (H2): Local government environmental confidence (LGC) positively predicts willingness to pay for environmental protection.*

*Hypothesis 3 (H3): The effect of LGC on WTP is stronger than that of CGC.*

### 2.3 Urban–rural heterogeneity in the confidence–WTP nexus

China’s urban–rural divide represents one of the most salient structural features of its social landscape. Yu (24) found that rural Chinese are less concerned about the environment than urban counterparts, especially regarding pollution, nature conservation, and global environmental degradation, partly due to limited education and information access. Chen et al. (41) found that respondents living in larger cities were more likely to participate in pro-environmental behavior. Cheng and Mao (25), using CGSS2021 data, demonstrated that urban residents generally exhibit better environmental behaviors, with education and income as critical drivers. Ding and Wang (26) found that the urban–rural divergence in WTP for green energy consumption stems primarily from the gap in capital endowment rather than energy cognition. Peng and Dan (42) documented significant digital divides between urban and rural areas in China that affect information access and economic outcomes.

We hypothesize that the relative importance of CGC versus LGC for WTP varies across urban and rural settings. Urban residents interact with local environmental governance more directly: they experience municipal waste management, witness industrial pollution control, and observe urban greening projects. For these residents, the quality of local environmental governance is tangible, making LGC a stronger predictor. Rural residents have less direct interaction with local environmental agencies and may perceive environmental governance as more of a top-down, policy-driven process. Major rural environmental improvements are widely understood as central government directives, and rural residents may rely more heavily on centrally controlled traditional media for environmental information (17, 24). Therefore:

*Hypothesis 4a (H4a): For urban residents, LGC is a significant positive predictor of WTP, while CGC is not significant.*

*Hypothesis 4b (H4b): For rural residents, CGC is a significant positive predictor of WTP, while LGC is not significant.*

### 2.4 Media use and willingness to pay for environmental protection

Media, as the primary channel through which the public acquires environmental information, has been extensively studied in relation to environmental attitudes and behaviors. Wang (10), adopting an environmental communication perspective, found that media use frequency significantly enhances WTP, mediated by individual environmental responsibility rather than environmental knowledge, and that traditional media use has a significant effect while new media use does not. Sun and Kong (11), analyzing CGSS2021 data, found that new media usage and media trust negatively impact WTP, attributing this to a ‘clicktivism’ mechanism whereby superficial online engagement substitutes for substantive economic commitment. Clicktivism, as conceptualized by Halupka (43), refers to political acts performed through simplified digital connectivity that have been marginalized through recurrent negative discourse. Christensen (44) found no evidence that Internet activism substitutes traditional participation, though the real-life impact of such activities remains debated.

The distinction between traditional and new media effects is particularly relevant in the Chinese media landscape. Hu et al. (18) confirmed that traditional media use is positively associated with environmental public service satisfaction, while new media use is negatively associated, with government trust mediating these relationships. Xiao et al. (45) found that Internet use has overall positive effects on pro-environmental behavior in China, particularly for private behaviors among low-income and female groups. Liu et al. (46) demonstrated that Internet use affects pro-environmental behaviors both directly and indirectly through environmental knowledge, perceived pollution threats, and government satisfaction. However, our preliminary analysis reveals that after controlling for government environmental confidence, the independent contribution of media use to explaining WTP variance is minimal, suggesting that media effects on WTP may operate partly through the government confidence channel.

*Hypothesis 5 (H5): Traditional media use frequency positively predicts WTP for environmental protection.*

*Hypothesis 6 (H6): New media use frequency negatively predicts WTP for environmental protection.*

### 2.5 Theoretical framework

**Figure 1:**
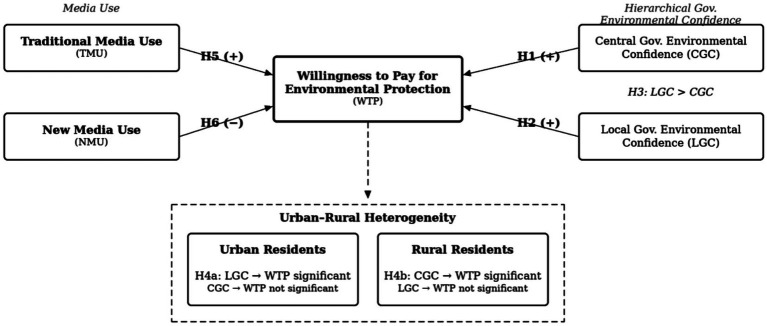
Conceptual model of dual-pathway influences on willingness to pay for environmental protection.

Integrating the above reasoning, this study proposes a dual-pathway model of environmental WTP. The first pathway posits that citizens’ evaluations of government environmental governance performance at different hierarchical levels independently shape WTP, with the relative importance of each level varying across urban and rural contexts. The second pathway posits that traditional and new media use exert direct, albeit modest, effects on WTP through information provision and behavioral orientation mechanisms. Drawing on the environmental communication tradition (47), this framework departs from previous studies that have sought to establish media-to-trust-to-behavior mediation chains (18, 28), recognizing that in the domain of environmental governance evaluation, the explanatory power of media use may be limited relative to citizens’ direct assessments of government performance. Figure 1 presents the conceptual model.

## 3 Research design

### 3.1 Data source

This study uses data from the 2021 Chinese General Social Survey (CGSS2021), a nationally representative survey initiated in 2003 by the National Survey Research Center at Renmin University of China. The CGSS employs a multi-stage stratified random sampling procedure covering 100 counties (districts) across 29 provinces, with four communities or villages randomly selected within each county, and 25 households surveyed per community. One eligible adult member per household is randomly selected for face-to-face interview. The CGSS2021 dataset comprises 8,148 valid respondents. The environmental module (ISSP Environment IV), which contains the key dependent and mediating variables used in this study, was administered to a subsample of 2,741 respondents as a rotating module. After listwise deletion of cases with missing values on all core variables (TMU, NMU, CGC, LGC, WTP, and control variables), the final analytical sample consists of 1,638 respondents. Comparison of the environmental module subsample (*N* = 2,741) and the final analytical sample reveals that the analytical sample is slightly older (*M* = 53.5 vs. 51.4, *p* < 0.001) and reports marginally higher WTP (*M* = 3.09 vs. 3.01, *p* = 0.007). Other variables show no significant differences (all *p* > 0.05), suggesting that these minor compositional differences are unlikely to substantively affect the core findings.

### 3.2 Variable measurement

#### 3.2.1 Dependent variable: willingness to pay for environmental protection (WTP)

WTP was measured using three items from the CGSS2021 ISSP module: P11a (‘To protect the environment, to what extent are you willing to pay higher prices?’), P11b (‘To protect the environment, to what extent are you willing to pay higher taxes?’), and P11c (‘To protect the environment, to what extent are you willing to accept a reduction in your standard of living?’). Each item was rated on a five-point scale (1 = very willing to 5 = very unwilling). We reverse-coded all items so that higher values indicate greater willingness (1 = very unwilling to 5 = very willing). A composite WTP index was computed as the mean of the three reverse-coded items (Cronbach’s *α* = 0.774, *M* = 3.090, SD = 0.941). This measurement approach is consistent with prior studies using CGSS data (9–11).

#### 3.2.2 Independent variables: hierarchical government environmental confidence

Central government environmental confidence (CGC) was measured using item H11: ‘In addressing China’s domestic environmental problems, how do you evaluate the central government’s performance over the past five years?’ Responses were coded on a five-point scale (1 = focused solely on economic development while neglecting environmental protection; 2 = insufficient attention with inadequate environmental investment; 3 = made efforts but with poor results; 4 = made great efforts with some achievements; 5 = achieved great accomplishments; *M* = 4.073, *SD* = 0.897).

Local government environmental confidence (LGC) was measured using item H8: ‘In addressing the environmental problems in your residential area, how do you evaluate the local government’s performance over the past five years?’ The response scale was identical to CGC (*M* = 3.745, *SD* = 0.980). A paired-samples *t*-test confirmed that CGC was significantly higher than LGC (*t* = 14.804, *p* < 0.001, mean difference = 0.328), consistent with the hierarchical government trust pattern documented in the literature (13–15). These items measure citizens’ retrospective performance evaluations, which we interpret as indicators of domain-specific environmental governance confidence following the conceptual framework outlined in Section 2.2.

#### 3.2.3 Independent variables: media use

The relatively low internal consistency of the four-item traditional media use (TMU) index (Cronbach’s α = 0.525; M = 2.152, SD = 0.663) reflects the heterogeneous nature of traditional media formats, which is a common finding in CGSS-based studies (10, 11); full measurement details are provided in the Supplementary material. Specifically, television remains widely used among older populations, whereas newspaper and magazine readership has declined substantially, resulting in divergent usage patterns across the four items. Despite the low *α*, we retain the composite index for comparability with prior studies. To address this concern, we report three robustness measures: (1) item-level descriptive statistics in Supplementary Table S4; (2) a three-item index excluding television (α = 0.625) with consistent results in Section 4.5; and (3) the core findings regarding government environmental confidence remain unchanged regardless of TMU operationalization.

New media use (NMU) was measured using the single item for Internet use frequency, including mobile Internet access (A28, sub-item 5; *M* = 3.303, *SD* = 1.664).

#### 3.2.4 Control variables

Following prior research (8–11, 25), we controlled for gender (male = 1; 48.2%), age (*M* = 53.494, *SD* = 16.215), education (years of schooling; *M* = 8.855, *SD* = 4.471), household income (natural log; *M* = 10.353, *SD* = 2.392), Communist Party membership (member = 1; 13.1%), urban/rural residence (urban = 1; 64.9%), environmental concern (P6, five-point scale; *M* = 3.645, *SD* = 0.923), and environmental severity perception (H1, five-point scale; *M* = 2.971, *SD* = 1.116).

### 3.3 Analytical strategy

We employed hierarchical multiple regression analysis with heteroskedasticity-robust standard errors (HC1). Four nested models were estimated: Model 1 (baseline, control variables only), Model 2 (adding media use variables), Model 3 (adding government environmental confidence variables), and Model 4 (full model with all predictors). This hierarchical approach allows us to assess the incremental variance explained by each set of predictors through changes in *R*^2^ (ΔR^2^). Urban–rural heterogeneity was examined through split-sample analysis. Robustness was assessed through: (a) replacing the four-item TMU with a three-item index excluding television; (b) adding social trust as an additional control; and (c) disaggregating WTP into its three constituent items. Additionally, we conducted bootstrap mediation analysis (5,000 resamples) to explore whether government environmental confidence mediates the media use–WTP relationship. All variance inflation factors (VIF) were below 2.0, indicating no multicollinearity concerns.

## 4 Results

### 4.1 Descriptive statistics

The descriptive results reveal a clear hierarchical confidence pattern: respondents rated the central government’s environmental performance (*M* = 4.073) significantly higher than the local government’s (*M* = 3.745; paired *t* = 14.804, *p* < 0.001) (Table 1). The mean WTP index (3.090) was slightly above the scale midpoint, with willingness to pay higher prices (3.303) exceeding willingness to pay higher taxes (3.120) and willingness to accept lower living standards (2.860). New media use (*M* = 3.303) was substantially more frequent than traditional media use (*M* = 2.152).

**Table 1:** Descriptive statistical results of variables.

Variable	*N*	Mean	Std. dev.	Min	Max
TMU	1,638	2.152	0.663	1	5
NMU	1,638	3.303	1.664	1	5
CGC	1,638	4.073	0.897	1	5
LGC	1,638	3.745	0.980	1	5
WTP	1,638	3.090	0.941	1	5
WTP-price	1,609	3.303	1.092	1	5
WTP-tax	1,605	3.120	1.136	1	5
WTP-living standard	1,617	2.860	1.146	1	5
Gender (male = 1)	1,638	0.482	0.500	0	1
Age	1,638	53.494	16.215	18	94
Education (years)	1,638	8.855	4.471	0	19
Income (ln)	1,638	10.353	2.392	0	16.1
Party member	1,638	0.131	0.337	0	1
Urban	1,638	0.649	0.477	0	1
Env. concern	1,638	3.645	0.923	1	5
Env. severity	1,638	2.971	1.116	1	5

### 4.2 Hierarchical regression analysis

The hierarchical regression results in Table 2 reveal several key findings. First, government environmental confidence variables (Model 3) contribute substantially more incremental variance (ΔR^2^ = 0.021) than media use variables (Model 2, ΔR^2^ = 0.002), indicating that government confidence is a far more powerful predictor of WTP than media use frequency.

**Table 2:** Hierarchical regression results (DV: WTP).

Variables	Model 1	Model 2	Model 3	Model 4	Model 5	Model 6
TMU	—	0.031 (0.039)	—	0.030 (0.039)	0.018 (0.039)	—
TMU3	—	—	—	—	—	0.046 (0.035)
NMU	—	−0.033* (0.020)	—	−0.033* (0.019)	−0.032* (0.019)	−0.034* (0.019)
CGC	—	—	0.061** (0.030)	0.062** (0.030)	0.059** (0.030)	0.063** (0.030)
LGC	—	—	0.106*** (0.028)	0.105*** (0.028)	0.099*** (0.028)	0.104*** (0.028)
Social trust	—	—	—	—	0.078*** (0.028)	—
Gender	0.074 (0.047)	0.065 (0.047)	0.071 (0.046)	0.062 (0.046)	0.068 (0.046)	0.061 (0.046)
Age	−0.003 (0.002)	−0.005** (0.002)	−0.003* (0.002)	−0.005*** (0.002)	−0.005*** (0.002)	−0.005*** (0.002)
Education	−0.007 (0.006)	−0.006 (0.006)	−0.006 (0.006)	−0.005 (0.006)	−0.005 (0.006)	−0.006 (0.006)
Income	0.004 (0.011)	0.005 (0.011)	0.004 (0.011)	0.005 (0.011)	0.006 (0.011)	0.004 (0.011)
Party member	0.305*** (0.068)	0.311*** (0.068)	0.287*** (0.066)	0.294*** (0.067)	0.268*** (0.067)	0.290*** (0.067)
Urban	0.013 (0.051)	0.024 (0.052)	0.016 (0.051)	0.028 (0.051)	0.036 (0.051)	0.026 (0.051)
Env. concern	0.210*** (0.028)	0.207*** (0.028)	0.194*** (0.028)	0.191*** (0.028)	0.188*** (0.028)	0.190*** (0.028)
Env. severity	−0.030 (0.022)	−0.029 (0.022)	0.001 (0.022)	0.001 (0.022)	0.005 (0.022)	0.001 (0.022)
R^2^	0.061	0.063	0.081	0.084	0.091	0.084
ΔR^2^ (vs M1)	—	0.002	0.021	0.023	0.031	0.023

Unstandardized coefficients. *p < 0.1; **p < 0.05; ***p < 0.01. Robust standard errors in parentheses. Model 5: *N* = 1,632; Model 6: TMU replaced by three-item index.

In the full model (Model 4), both CGC (*b* = 0.062, *p* < 0.05) and LGC (*b* = 0.105, *p* < 0.01) significantly and positively predict WTP, supporting H1 and H2. The coefficient of LGC is numerically larger than that of CGC; however, a formal Wald test for coefficient equality does not reach statistical significance (*t* = −0.867, *p* = 0.386), and thus H3 is not supported. For media use, TMU shows no significant effect on WTP (*b* = 0.030, *p* = 0.447), failing to support H5. NMU shows a significant negative effect, supporting H6.

Among control variables, Communist Party membership (*b* = 0.294, *p* < 0.01) and environmental concern (*b* = 0.191, *p* < 0.01) are the strongest positive predictors of WTP, while age shows a significant negative effect (*b* = −0.005, *p* < 0.01). Notably, environmental severity perception becomes non-significant once government confidence is controlled, suggesting that severity perception operates on WTP primarily through its influence on government evaluation.

### 4.3 Disaggregated WTP analysis

The disaggregated analysis (Table 3) reveals that LGC is the most consistently significant predictor, reaching significance across all three WTP dimensions (price: *b* = 0.111, *p* < 0.01; tax: *b* = 0.100, *p* < 0.01; living standard: *b* = 0.089, *p* < 0.05). CGC reaches significance for the tax dimension (*b* = 0.063, *p* < 0.1) and the living standard dimension (*b* = 0.086, *p* < 0.05), suggesting that central government confidence matters more for higher-cost forms of environmental sacrifice. NMU’s negative effect is concentrated exclusively in the living standard dimension (*b* = −0.066, *p* < 0.01), consistent with the interpretation that new media’s clicktivism effect is primarily associated with lower willingness to make substantial lifestyle sacrifices.

**Table 3:** Regression results by WTP dimension (Full Model specification).

Variables	WTP-price	WTP-tax	WTP-living std.
TMU	0.038 (0.044)	0.048 (0.045)	−0.000 (0.044)
NMU	−0.018 (0.022)	−0.028 (0.023)	−0.066*** (0.024)
CGC	0.035 (0.036)	0.063* (0.036)	0.086** (0.038)
LGC	0.111*** (0.034)	0.100*** (0.036)	0.089** (0.035)
Gender	0.077 (0.054)	0.084 (0.056)	0.020 (0.057)
Age	−0.005** (0.002)	−0.007*** (0.002)	−0.004* (0.002)
Education	−0.005 (0.007)	−0.008 (0.008)	−0.001 (0.008)
Income	0.004 (0.013)	0.007 (0.013)	0.004 (0.013)
Party member	0.328*** (0.076)	0.296*** (0.083)	0.240*** (0.082)
Urban	0.034 (0.060)	0.037 (0.062)	0.009 (0.063)
Env. concern	0.209*** (0.032)	0.181*** (0.034)	0.172*** (0.035)
Env. severity	0.003 (0.026)	−0.000 (0.027)	0.001 (0.028)
*R* ^2^	0.080	0.070	0.046

Unstandardized coefficients. *p < 0.1; **p < 0.05; ***p < 0.01. Robust standard errors in parentheses. Control variables included.

### 4.4 Urban–rural subgroup analysis

The subgroup analysis reveals the study’s most notable finding, supporting H4a and H4b in the subgroup specification (Table 4). For urban residents (*N* = 1,063), LGC is a highly significant predictor of WTP (*b* = 0.132, *p* < 0.01), while CGC is not significant (*b* = 0.033, *p* = 0.377). The pattern reverses for rural residents (*N* = 575): CGC significantly predicts WTP (*b* = 0.116, *p* < 0.05), while LGC does not (*b* = 0.056, *p* = 0.221). This pattern suggests that urban residents base their WTP decisions on local government performance assessments, whereas rural residents rely on their evaluation of the central government’s environmental commitment.

**Table 4:** Subgroup regression results by urban/rural residence (DV: WTP).

Variables	Urban (*N* = 1,063)	Rural (*N* = 575)
TMU	0.005 (0.047)	0.090 (0.068)
NMU	−0.017 (0.023)	−0.054 (0.035)
CGC	0.033 (0.037)	0.116** (0.050)
LGC	0.132*** (0.039)	0.056 (0.045)
Gender	0.066 (0.057)	0.044 (0.077)
Age	−0.004* (0.002)	−0.007** (0.003)
Education	−0.006 (0.008)	0.001 (0.010)
Income	0.006 (0.014)	0.001 (0.017)
Party member	0.305*** (0.079)	0.264** (0.122)
Env. concern	0.199*** (0.033)	0.170*** (0.051)
Env. severity	0.011 (0.027)	−0.016 (0.038)
R^2^	0.076	0.112

Unstandardized coefficients. *p < 0.1; **p < 0.05; ***p < 0.01. Robust standard errors in parentheses. Urban/rural variable excluded.

The overall model fit is substantially better for the rural subsample (R^2^ = 0.112) than for the urban subsample (R^2^ = 0.076), suggesting that the variables included in our model explain a larger portion of WTP variance in rural areas. Media use variables are non-significant in both subsamples. To formally test whether the urban–rural differences are statistically significant, we estimated an interaction model including CGC × Urban and LGC × Urban terms. The interaction coefficients are directionally consistent with the subgroup pattern (CGC × Urban = −0.069, *p* = 0.275; LGC × Urban = 0.079, *p* = 0.166) but do not reach conventional significance levels. This suggests that the urban–rural divergence, while robust in subgroup analysis, should be interpreted as a suggestive rather than definitive pattern.

### 4.5 Robustness checks

Several robustness checks were conducted. First, as WTP items are ordinal in nature, we re-estimated the disaggregated models using ordered logistic regression (Table A1). The results largely confirm the core OLS findings: CGC and LGC remain positive and highly significant across all three dimensions (all *p* < 0.01), and NMU remains significantly negative (all *p* < 0.01). TMU shows positive significance for the price and tax dimensions in the ordered logit specification, suggesting that the OLS null finding for TMU should be interpreted with some caution. The ordered logit results are generally stronger than OLS, reinforcing the robustness of the core conclusions. Second, replacing the four-item TMU with a three-item index excluding television (Model 6 in Table 2) produces consistent results: CGC (*b* = 0.063, *p* < 0.05) and LGC (*b* = 0.104, *p* < 0.01) remain significant. Third, adding social trust as an additional control (Model 5) does not alter the significance of CGC (*b* = 0.059, *p* < 0.05) or LGC (*b* = 0.099, *p* < 0.01); social trust itself is a significant positive predictor (*b* = 0.078, *p* < 0.01), consistent with research on social capital and environmental behavior (48). Fourth, all VIF values are below 2.0 (range: 1.05–1.99), confirming the absence of multicollinearity.

Finally, although our theoretical framework posits parallel rather than sequential pathways, we conducted exploratory bootstrap mediation analysis (5,000 resamples) to examine whether government environmental confidence mediates the media use–WTP relationship. None of the four indirect paths (TMU/NMU → CGC/LGC → WTP) reached significance, with all 95% bootstrap confidence intervals containing zero. These null mediation results confirm that media use and government environmental confidence function as parallel, independent pathways to WTP, which is consistent with the parallel-pathway specification adopted in this study.

### 4.6 Summary of hypothesis testing

Table 5 summarizes the results of hypothesis testing. Of the seven hypotheses, H1, H2, and H6 are supported: both central and local government environmental confidence positively predict WTP, and new media use negatively predicts WTP. H3 is not supported, as the difference between the LGC and CGC coefficients does not reach statistical significance. H4a and H4b are partially supported: the expected urban–rural pattern holds in the subgroup analysis, but the corresponding interaction terms do not reach conventional significance levels. H5 is not supported, as traditional media use shows no significant effect on WTP in the main models.

**Table 5:** Summary of hypothesis testing results.

	Hypothesis	Expected	Coefficient (S.E.)	Result
H1	CGC positively predicts WTP	(+)	0.062** (0.030)	Supported
H2	LGC positively predicts WTP	(+)	0.105*** (0.028)	Supported
H3	LGC effect > CGC effect		0.105 vs. 0.062	Not supported
H4a	Urban: LGC sig., CGC n.s.		0.132***/0.033	Partially supported
H4b	Rural: CGC sig., LGC n.s.		0.116**/0.056	Partially supported
H5	TMU positively predicts WTP	(+)	0.030 (0.039)	Not supported
H6	NMU negatively predicts WTP	(−)	−0.033* (0.019)	Supported

Coefficients from Model 4 (full model). *p < 0.1; **p < 0.05; ***p < 0.01. Robust standard errors in parentheses.

## 5 Discussion

### 5.1 Hierarchical government environmental confidence as a predictor of WTP

The most robust finding of this study is that both central and local government environmental confidence significantly and positively predict willingness to pay for environmental protection. Local government confidence (LGC) shows a numerically larger coefficient than central government confidence (CGC). These results are consistent across all model specifications and robustness checks, confirming H1 and H2. H3, however, is not supported, as the coefficient difference does not reach statistical significance.

This finding extends the existing literature in two important ways. First, while prior studies have established that institutional trust broadly promotes environmental WTP (20, 22, 49), our study demonstrates that this relationship operates differentially across government levels. Tan et al. (20) treated trust as a single construct; Anderson (22) confirmed a positive trust-WTP relationship without distinguishing government levels. By introducing the hierarchical dimension, we show that confidence in local implementation shows a numerically larger association with WTP than confidence in national strategy, though the difference is not statistically significant.

Second, the numerically larger coefficient of LGC, while not statistically distinguishable from CGC, is nonetheless theoretically suggestive. Environmental protection is a domain where policy implementation occurs primarily at the local level (31, 32). When citizens evaluate whether their financial contributions will produce tangible improvements, they are essentially judging local government capacity. This aligns with Zhai’s (36) finding that both government levels lose trust for poor environmental performance, and with Huang’s (37) observation that Chinese citizens attribute implementation problems to local authorities.

Notably, environmental severity perception loses its direct effect on WTP once government confidence is controlled (from *b* = −0.030, *p* = 0.182 in Model 1 to *b* = 0.001, *p* = 0.953 in Model 4). Supplementary analysis suggests that severity is negatively associated with both CGC and LGC. This suggests that perceived environmental severity does not exhibit a significant independent association with WTP in the main models, but may be indirectly related to WTP through its negative association with government confidence in government environmental governance (11). Citizens who perceive severe environmental problems may develop lower confidence in government environmental efforts, which in turn may be associated with reduced willingness to invest personal resources.

### 5.2 The urban–rural divergence

The urban–rural subgroup analysis reveals a suggestive pattern. For urban residents, WTP is more closely associated with local government confidence (*b* = 0.132, *p* < 0.01), while CGC is non-significant. For rural residents, the pattern reverses: WTP is more closely associated with central government confidence (*b* = 0.116, *p* < 0.05), while LGC is non-significant. This pattern supports H4a and H4b in the subgroup analysis, though the interaction terms in the full-sample model do not reach conventional significance levels, suggesting this finding should be interpreted with caution.

This divergence can be explained through three complementary mechanisms. First, urban residents have more direct interactions with local environmental governance. Municipal waste management, industrial emission control, and urban greening are visible features of urban life (24, 25). Rural residents, by contrast, may perceive environmental governance as a top-down process, with improvements understood as central government directives (24, 32). Second, information environments differ systematically. Xu et al. (17) demonstrated that traditional media is associated with higher central government trust while social media is associated with lower local government trust. Rural residents rely more heavily on television (24, 26), so their confidence assessments may be shaped by centrally curated narratives. Third, this pattern resonates with the hierarchical trust literature: Li (15) showed rural villagers trust the central government’s intent while distrusting local officials; Chen (39) found that public service quality predicts local but not central trust.

From a practical standpoint, campaigns aimed at increasing urban residents’ WTP should emphasize local government environmental achievements and demonstrate local accountability. In rural areas, effective messaging should highlight the central government’s environmental commitment and connect personal contributions to national goals.

### 5.3 The role of media use

Our finding that traditional media use shows no significant effect on WTP (H5 not supported) while new media use shows a significant negative effect (*b* = −0.033, *p* < 0.1, H6 supported) provides important nuance to the environmental communication literature.

These results both converge with and diverge from prior findings. Wang (10) found that traditional media use significantly increases WTP; Sun and Kong (11) found that new media negatively affects WTP. The key difference is that these studies did not control for government environmental confidence. Huang (50), examining internet usage and environmental governance satisfaction using Chinese survey data, similarly found that media use shapes environmental perceptions but did not incorporate hierarchical government confidence into the analytical framework. Our results suggest that what Wang (10) attributed to media’s responsibility-cultivation function may partly reflect media’s role in shaping government evaluations—consistent with Hu et al.’s (18) finding that government trust mediates the media-satisfaction relationship. When government confidence is explicitly modeled, the residual direct effect of traditional media diminishes to non-significance.

The negative effect of new media on WTP is concentrated in the living standard dimension (*b* = −0.066, *p* < 0.01). This finding aligns with the clicktivism interpretation, whereby online engagement may serve as a psychological substitute for more costly forms of environmental commitment (43, 44). Notably, this effect does not extend to less costly dimensions (prices, taxes), suggesting that clicktivism is specifically associated with lower willingness for substantial lifestyle changes.

### 5.4 Theoretical contributions

This study makes three theoretical contributions. First, it extends the hierarchical government trust framework (13–16) into the environmental WTP domain, demonstrating that citizens’ differential evaluations of central versus local government performance have distinct implications for environmental investment decisions.

Second, it provides suggestive evidence of urban–rural heterogeneity in the confidence–WTP relationship, indicating that urban residents’ WTP appears more closely linked to local government evaluations while rural residents’ WTP appears more closely linked to central government evaluations. This enriches research on urban–rural environmental behavior disparities (24–26).

Third, it suggests that, under the current operationalization, government environmental confidence accounts for substantially more WTP variance than media use frequency. This recalibrates the emphasis in environmental communication research (10, 11, 46) by showing that citizens’ direct evaluations of government performance are substantially more consequential for WTP.

## 6 Conclusion

### 6.1 Summary of findings

This study examined how media use and hierarchical government environmental confidence jointly predict WTP among Chinese residents, using CGSS2021 data (*N* = 1,638). The key findings are as follows. First, both CGC (*b* = 0.062, *p* < 0.05) and LGC (*b* = 0.105, *p* < 0.01) positively predict WTP, with LGC showing a numerically larger coefficient, though the difference is not statistically significant. Second, subgroup analysis suggests an urban–rural divergence: urban residents’ WTP is more closely associated with local government confidence, whereas rural residents’ WTP is more closely associated with central government confidence. Third, new media use is negatively associated with WTP, while traditional media use has no significant effect. Government confidence variables explain substantially more WTP variance than media use variables.

### 6.2 Policy recommendations

First, local governments should enhance the visibility and transparency of environmental governance outcomes, as local government confidence is the strongest predictor of WTP overall and the dominant predictor for urban residents. Regular publication of environmental monitoring data and public disclosure of environmental fund allocation can strengthen public confidence.

Second, environmental communication strategies should be differentiated by context. For urban audiences, messaging should emphasize local achievements and measurable improvements. For rural audiences, communication should highlight the central government’s policy commitments and national ecological civilization targets.

Third, given the negative effect of new media use on WTP for higher-cost sacrifices, environmental communicators should develop strategies that channel online engagement toward substantive economic participation rather than allowing it to serve as a substitute.

Fourth, the strong positive effect of Communist Party membership on WTP (*b* = 0.294, *p* < 0.01) suggests that grassroots party organizations can play an important role in environmental mobilization through community-level initiatives and demonstration effects.

### 6.3 Limitations and future directions

First, the cross-sectional nature of the CGSS2021 data precludes causal inference. While our theoretical framework posits that government environmental confidence influences WTP, the reverse causal direction cannot be ruled out: citizens who are more willing to invest in environmental protection may develop more favorable evaluations of government environmental performance. Additionally, the data capture attitudes at a single point in time and cannot account for how government confidence may evolve dynamically in response to policy changes, environmental events, or shifts in media coverage. Future research using panel data, longitudinal designs, or natural experiments could help establish causal directionality and examine temporal dynamics in the confidence–WTP relationship. Second, government environmental confidence relies on single-item indicators, limiting the ability to model these as latent variables. Future surveys could develop multi-item scales. Third, the traditional media use index has relatively low internal consistency (*α* = 0.525), although robustness checks produce consistent results. Fourth, the dichotomous urban–rural classification may obscure within-category heterogeneity. Fifth, WTP as a stated preference measure may overestimate actual payment behavior. Despite these limitations, this study provides the first empirical evidence that hierarchical government environmental confidence differentially predicts WTP across urban and rural China.

## Data Availability

The original contributions presented in the study are included in the article/Supplementary material, further inquiries can be directed to the corresponding author.

## Appendix A

**Table A1:** Ordered logistic regression results by WTP dimension (robustness check).

Variables	WTP-price	WTP-tax	WTP-living std.
TMU	0.069*** (0.020)	0.068*** (0.020)	−0.019 (0.020)
NMU	−0.037*** (0.012)	−0.046*** (0.012)	−0.071*** (0.012)
CGC	0.093*** (0.011)	0.126*** (0.011)	0.200*** (0.011)
LGC	0.189*** (0.012)	0.171*** (0.012)	0.175*** (0.012)

Unstandardized coefficients from ordered logistic regression. **p* < 0.1; ***p* < 0.05; ****p* < 0.01. Standard errors in parentheses. Control variables included.
