# Supplementary material for: Hierarchical government environmental confidence and willingness to pay for environmental protection: evidence from urban–rural China
Source: Front Public Health. 2026 Jun 19;14:1853242. doi: 10.3389/fpubh.2026.1853242 (PMC13328507; doi:10.3389/fpubh.2026.1853242)
Supplement: Supplementary file 1 [file Table_1.docx]

**Supplementary Material: Variable Definitions and Coding Scheme**

*Study: Hierarchical Government Environmental Confidence and Willingness to Pay for Environmental Protection*

*Data Source: Chinese General Social Survey 2021 (CGSS2021) | Analytical Sample: N = 1,638*

**1. Dependent Variable**

**Table S1. Dependent variable: Willingness to Pay for Environmental Protection (WTP)**

| **Role** | **Variable** | **CGSS Item** | **Item Wording** | **Coding and Operationalization** |
| --- | --- | --- | --- | --- |
| DV | WTP-price | P11a | To protect the environment, to what extent are you willing to pay higher prices? | 1–5 (reverse-coded: 6 minus original). 1 = very unwilling, 5 = very willing |
| DV | WTP-tax | P11b | To protect the environment, to what extent are you willing to pay higher taxes? | Same as above |
| DV | WTP-living | P11c | To protect the environment, to what extent are you willing to accept a reduction in your standard of living? | Same as above |
| DV | WTP (index) | P11a–c | Composite index | Mean of three reverse-coded items. Cronbach’s α = 0.774 |

**2. Independent Variables: Hierarchical Government Environmental Confidence**

**Table S2. Government environmental confidence variables**

| **Role** | **Variable** | **CGSS Item** | **Item Wording** | **Coding and Operationalization** |
| --- | --- | --- | --- | --- |
| IV | CGC | H11 | In addressing China’s domestic environmental problems, how do you evaluate the central government’s performance over the past five years? | 1 = focused solely on economic development; 2 = insufficient attention; 3 = made efforts but poor results; 4 = great efforts with achievements; 5 = great accomplishments. M = 4.073, SD = 0.897 |
| IV | LGC | H8 | In addressing the environmental problems in your residential area, how do you evaluate the local government’s performance over the past five years? | Same scale as CGC. M = 3.745, SD = 0.980. Paired t-test: CGC > LGC (t = 14.804, p < 0.001) |

*Note: CGC = Central Government Environmental Confidence; LGC = Local Government Environmental Confidence. Values ≥ 98 coded as missing.*

**3. Independent Variables: Media Use**

**Table S3. Media use variables**

| **Role** | **Variable** | **CGSS Item** | **Item Wording** | **Coding and Operationalization** |
| --- | --- | --- | --- | --- |
| IV | TMU | A28_1–4 | In the past year, how often did you use the following media? (a) Newspaper; (b) Magazine; (c) Radio; (d) Television | 1 = never, 2 = rarely, 3 = sometimes, 4 = often, 5 = very frequently. Composite: mean of four items. α = 0.525. Robustness: TMU3 excludes TV (α = 0.625) |
| IV | NMU | A28_5 | In the past year, how often did you use the Internet (including mobile Internet)? | Same 1–5 scale. Single item. M = 3.303, SD = 1.664 |

*Note: TMU = Traditional Media Use; NMU = New Media Use. Values ≥ 98 coded as missing.*

**4. Control Variables**

**Table S4. Control variables**

| **Role** | **Variable** | **CGSS Item** | **Item Wording** | **Coding and Operationalization** |
| --- | --- | --- | --- | --- |
| Control | Gender | A2 | What is your gender? | 0 = female, 1 = male |
| Control | Age | A3_1 | Year of birth | 2021 minus birth year (continuous) |
| Control | Education | A7a | What is your highest level of education? | Converted to years of schooling: no education = 0, primary = 6, junior high = 9, senior high/vocational = 12, junior college = 15, bachelor’s = 16, graduate = 19 |
| Control | Income (ln) | A62 | What was your total personal income last year (RMB)? | Natural logarithm of (income + 1). Values ≥ 9,999,996 coded as missing |
| Control | Party member | A10 | Are you a member of the Communist Party of China? | 1 = CPC member (A10 = 4), 0 = otherwise. Values ≥ 98 = missing |
| Control | Urban/Rural | isurban | Urban or rural residence | 1 = urban, 0 = rural |
| Control | Env. concern | P6 | How concerned are you about environmental issues? | 1–5 scale (higher = more concerned). Values ≥ 98 = missing |
| Control | Env. severity | H1 | How serious do you think China’s environmental problems are? | 1–5 scale (higher = more severe). Values ≥ 98 = missing |

**5. Robustness Check Variables**

**Table S5. Additional variables for robustness checks**

| **Role** | **Variable** | **CGSS Item** | **Item Wording** | **Coding and Operationalization** |
| --- | --- | --- | --- | --- |
| Robust | TMU3 | A28_1–3 | Newspaper + Magazine + Radio (excluding TV) | Mean of three items. α = 0.625 |
| Robust | Social trust | A33 | Generally speaking, do you think most people can be trusted? | 1–5 scale. Values ≥ 98 = missing |

**6. Sample Construction**

Step 1: Full CGSS2021 sample: N = 8,148

Step 2: Environmental module (ISSP rotating module) subsample: N = 2,741

Step 3: Listwise deletion of missing values on all core variables (TMU, NMU, CGC, LGC, WTP, and 8 control variables)

**Final analytical sample: N = 1,638**

*Missing data diagnostics: The analytical sample is slightly older (M = 53.5 vs. 51.4, p < 0.001) and reports marginally higher WTP (M = 3.09 vs. 3.01, p = 0.007) than the full environmental module subsample. Other variables show no significant differences (all p > 0.05).*
